# Tissue and serum samples of patients with papillary thyroid cancer with and without benign background demonstrate different altered expression of proteins

**DOI:** 10.7717/peerj.2450

**Published:** 2016-09-13

**Authors:** Mardiaty Iryani Abdullah, Ching Chin Lee, Sarni Mat Junit, Khoon Leong Ng, Onn Haji Hashim

**Affiliations:** 1 Department of Molecular Medicine, Faculty of Medicine, University of Malaya, Kuala Lumpur, Malaysia; 2 University of Malaya Centre for Proteomics Research, Faculty of Medicine, University of Malaya, Kuala Lumpur, Malaysia; 3 Department of Surgery, Faculty of Medicine, University of Malaya, Kuala Lumpur, Malaysia

**Keywords:** Papillary thyroid cancer, Tissue proteomics, Serum proteomics, Benign thyroid goiter, Tissue alpha-1 antitrypsin, Alpha 2-HS glycoprotein, Biomarkers

## Abstract

**Background:**

Papillary thyroid cancer (PTC) is mainly diagnosed using fine-needle aspiration biopsy. This most common form of well-differentiated thyroid cancer occurs with or without a background of benign thyroid goiter (BTG).

**Methods:**

In the present study, a gel-based proteomics analysis was performed to analyse the expression of proteins in tissue and serum samples of PTC patients with (PTCb; n = 6) and without a history of BTG (PTCa; n = 8) relative to patients with BTG (n = 20). This was followed by confirmation of the levels of proteins which showed significant altered abundances of more than two-fold difference (*p* < 0.01) in the tissue and serum samples of the same subjects using ELISA.

**Results:**

The data of our study showed that PTCa and PTCb distinguish themselves from BTG in the types of tissue and serum proteins of altered abundance. While higher levels of alpha-1 antitrypsin (A1AT) and heat shock 70 kDa protein were associated with PTCa, lower levels of A1AT, protein disulfide isomerase and ubiquitin-conjugating enzyme E2 N seemed apparent in the PTCb. In case of the serum proteins, higher abundances of A1AT and alpha 1-beta glycoprotein were detected in PTCa, while PTCb was associated with enhanced apolipoprotein A-IV and alpha 2-HS glycoprotein (AHSG). The different altered expression of tissue and serum A1AT as well as serum AHSG between PTCa and PTCb patients were also validated by ELISA.

**Discussion:**

The distinctive altered abundances of the tissue and serum proteins form preliminary indications that PTCa and PTCb are two distinct cancers of the thyroid that are etiologically and mechanistically different although it is currently not possible to rule out that they may also be due other reasons such as the different stages of the malignant disease. These proteins stand to have a potential use as tissue or serum biomarkers to discriminate the three different thyroid neoplasms although this requires further validation in clinically representative populations.

## Introduction

Papillary thyroid cancer (PTC) is one of the few cancers whose incidence is on the rise (Enewold et al., 2009; Raposo et al., 2016). It is a follicular cell-derived cancer and accounts for 80% of all thyroid malignancies (Zhu et al., 2009). Most patients with PTC are considered to be of low risk (Garas et al., 2013), with 99% survival at 20 years after surgery (Shaha, Shah & Loree, 1997; Kakudo et al., 2004). Status of malignancy of the patients can be confirmed or nullified by a fine needle aspiration biopsy (FNAB) and followed by standard cytopathologic diagnosis. However, approximately 10–20% of the FNAB cytopathologic diagnosis results are inconclusive (Chow et al., 2001), which leads to unnecessary thyroidectomy in some of the patients (Akgül et al., 2010). FNAB has low predictive value on malignancy in patients with benign lesions due to the presence of multiple nodules (Luo et al., 2012). Ultrasound features, such as hypoechoic appearance, microcalcifications, irregular borders, and increased vascularity, are increasingly in used to help distinguish malignancy in PTC patients with benign goiter (Kim et al., 2008).

Having a background of benign lesions is apparently common amongst patients with PTC (Gandolfi et al., 2004; Pang & Chen, 2007; Wang et al., 2013). Although multinodular goiter (MNG) was traditionally thought to be at a low risk for malignancy as compared to its single-nodule, numerous studies have reported a significant risk (Gandolfi et al., 2004; Smith et al., 2013). In Malaysia, 60% of patients who were diagnosed with thyroid cancers from years 1994 to 2004 were reported to have occurred with a background of prolonged goiter (Othman, Omar & Naing, 2009). In general, risk factors for malignancy in a benign lesion included female gender, older age (mean age was 47 years), multiple lesions and smaller nodule size (average diameter was 4 mM). It is believed that the prognosis of PTC with benign lesions is good, the incidence of recurrence is low and none of the patients died of cancer (Wang et al., 2013).

A significant number of proteomics analyses of tissue and/or blood samples from patients with PTC and those with benign thyroid goiter (BTG) have been reported. Srisomsap et al. (2002) have demonstrated increased tissue expression of ATP synthase D chain and prohibitin in a group of patients with PTC, whilst Brown et al. (2006) noted differentially expressed S100A6 protein (an isoform of S100 protein), peroxiredoxin 2 and heat shock protein 70 (HSP70). By using surface-enhanced laser desorption/ionization time-of-flight mass spectrometry technique, Fan et al. (2009) explored the serum protein profiles of patients with PTC and confirmed valuable biomarkers for thyroid diagnosis such as haptoglobulin alpha-1 chain, apolipoprotein C-I and apolipoprotein C-III. However, to date, comparative studies of the expression of proteins in the tissue and serum samples of PTC patients with and without benign background have not been performed. This is important as the two types of PTC maybe totally unrelated, both etiologically as well as mechanistically.

In the present study, we have compared the thyroid tissue and serum samples of patients with PTC without a history of BTG, which we termed PTCa, and PTC patients with a background of BTG, termed PTCb, relative to those expressed in patients with BTG, using gel-based proteomic analysis. Differences in the profiles of protein abundances in PTCa and PTCb relative to BTG would form a preliminary reflection that the two thyroid cancers are etiologically and mechanistically different.

## Materials and Methods

### Sample collection and processing

The study was conducted according to the declaration of Helsinki and approval was granted from the Medical Ethics Committee of University of Malaya Medical Centre (Reference number, 925.8). Informed written consent was obtained from BTG (n = 20), PTCa (n = 8) and PTCb (n = 6) patients, prior to collection of samples. Tissue specimens were collected from surgically removed thyroid lobes of the same patients subjected to either partial or total thyroidectomy. At the definitive histopathological examination, seven cases of unifocal PTC and one multifocal PTC involving both lobes were reported for the patients with PTCa, whilst all patients with PTCb showed unifocal tumors. Among the 20 BTG tissue specimens, 19 showed MNG and one sample was with multinodular colloid goiter. The samples were immersed in Allprotect Tissue Reagent (Qiagen, Germany) immediately after excision. Tissue protein was extracted from the patient’s thyroid tissue sample using Qiagen AllPrep DNA/RNA/Protein mini kit according to the manufacturer’s protocol. Pre-operative blood samples were obtained by peripheral venous puncture and collected into 1.5 ml BD vacutainers plain tubes (Becton, Dickinson & Co, Franklin Lakes, New Jersey, USA). The blood samples were immediately centrifuged at 3,000 × g for 15 min. The sera in the upper layer were stored in aliquots at −80 °C until used.

### Two-dimensional electrophoresis (2DE)

The protein pellet from thyroid tissue was dissolved in 200 μl of thiourea rehydration solution (7 M urea, 2 M thiourea, 2% w/v CHAPS, 0.4% v/v pH 4–7 IPG buffer). One hundred ng of dissolved tissue protein was then topped up to 200 μl with sample buffer (7 M urea, 2 M thiourea, 4% w/v CHAPS, 4% v/v pH 4–7 IPG buffer, 40 mM DTT, Orange G) and left at room temperature for 30 min. In case of serum samples, 7 μl was mixed with 21 μl sample buffer (9 M urea, 60 mM DTT, 2% (v/v) IPG buffer, 0.5% (v/v) Triton X-100) and incubated at room temperature for 30 min. The sample mixture was then made up to 200 μl by adding 172 μl rehydration buffer (8 M urea, 0.5% (v/v) IPG buffer and 0.5% (v/v) Triton X-100) and left for another 30 min at room temperature. Each rehydrated IPG Immobiline Drystrips pH 4–7, 11 cm (GE Healthcare, Uppsala, Sweden) was passively rehydrated overnight. Isoelectric focusing (IEF) of tissue and serum proteins was performed at 18 °C using the following program: 1) 500 V, 1 h, step and hold; 2) 1,000 V, 1 h, gradient; 3) 8,000 V, 2.5 h, gradient and 4) 8,000 V, 2.5 h, step and hold. Focused strips were equilibrated in 1.5 M Tris-HCl (pH 8.8) solution (6 M urea, 2% w/v SDS, 30% v/v glycerol, and 0.06 M DTT) for 15 min on a platform shaker, and further incubated in a similar equilibration solution containing 4.5% v/v iodoacetamide for another 15 min. The equilibrated strips were then overlaid onto 8–13% gradient polyacrylamide gels and electrophoresis was completed following an optimized protocol (Phase 1: 50 V, 40 mA, 25 W for 40 min; Phase 2: 600 V, 40 mA, 25 W for ∼2.5 h) using the SE 600 Ruby Electrophoresis System and Power Supply-EPS601 (GE Healthcare).

After electrophoresis, gels were stained according to the method described by Heukeshoven & Dernick (1988). Two-dimentional silver stained gels were digitalized on ImageScanner (GE HealthCare, Uppsala, Sweden) which were than analyzed using the ImageMaster 2D Platinum V 7.0 software (GE HealthCare, Uppsala, Sweden). Protein abundance was analyzed in terms of percentage of volume contribution, which is the volume of a protein spot expressed as a percentage of the total spot volume of all detected proteins. Protein spots showing at least a 1.5-fold difference in average expression level were considered statistically significant (*p* ≤ 0.01) and excised for identification by mass spectrometry.

### Trypsin digestion and mass spectrometry

Differentially expressed protein spots were manually excised from the 2DE gels. In-gel digestion with trypsin and analysis using Agilent 6550 iFunnel QTOF LC/MS system (Agilent, Santa Clara, CA, USA) were performed as earlier described by Lee et al. (2016).

### Database search

Spectrum Mill software (Agilent, Santa Clara, CA, USA) was set to search MS/MS acquired data against Swiss-Prot Homo sapiens database. Mass-tolerance of precursor and product ions was set to ± 20 and ± 50 ppm, respectively while carbamidomethylation was specified as a fixed modification and oxidized methionine as a variable modification. A protein was considered identified according to the following selection parameters: 1) Protein score specified to be more than 20; 2) peptide mass error less than 5 ppm; 3) forward-reverse score more than two; 4) peptide score more than six and 5) Scored Peak Intensity (%SPI) more than 60 percent.

### Enzyme-linked immunosorbent assay (ELISA)

All the collected serum specimens were analyzed by ELISA according to the manufacturers’ instructions. ELISA was performed using antihuman alpha-1 antitrypsin (A1AT), alpha 2-HS glycoprotein (AHSG), heat shock 70 kDa protein 1A (HSP70) as primary antibodies. Cut-off parameters for tissue and serum proteins selected for ELISA in both groups of PTCa and PTCb patients were: (1) fold change (f.c.) > 2.0 and (2) *p* < 0.01. ELISA kit for HSP70 (E3015Hu) was obtained from the Bioassay Technology Laboratory, Shanghai, China. Kits for estimation of A1AT (ab108798) and AHSG (ab108855) were purchased from Abcam®, Cambridge, UK. All readings were made on an ELISA Plate Reader (Bio-Rad, Hercules, CA, USA). All samples, standards and blanks were analyzed in duplicate.

### Statistical analysis

One-way ANOVA with Tukey’s post test was performed using GraphPad Prism version 5.00 for Windows (GraphPad Software, San Diego, California, USA). The ANOVA test was used to analyze the significance difference between the tissue or serum proteins of patients with PTCa or PTCb, relative to those with BTG. All values are expressed as mean ± standard error of the mean (SEM). A *p* value of less than 0.05 was considered significant.

## Results

**Figure 1:**
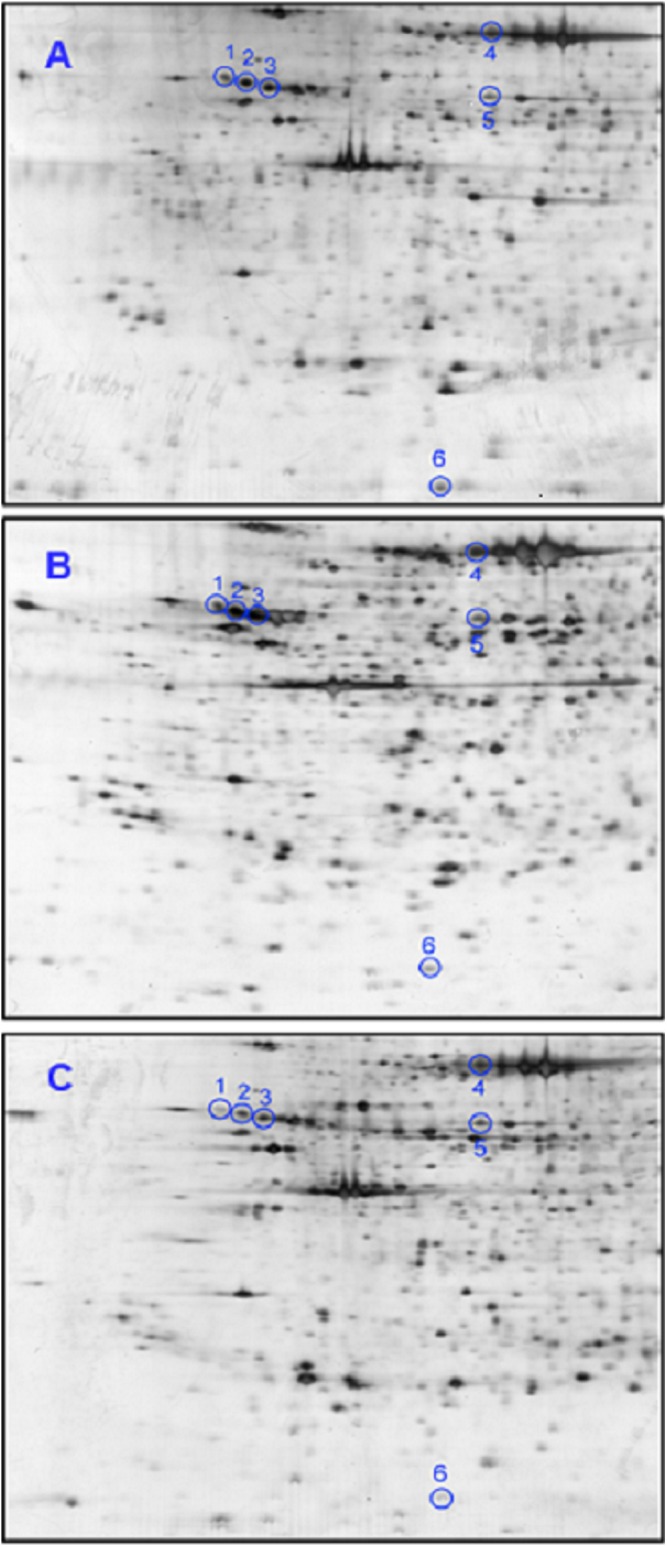
Representative 2DE tissue protein profiles of BTG, PTCa and PTCb patients. Tissue samples of (A) BTG (n = 20), (B) PTCa (n = 8) and (C) PTCb (n = 6) patients were subjected to 2DE and silver staining. Spots circled in blue are those that showed significant differential abundance in PTCa and/or PTCb patients, relative to patients with BTG. For all panels, the acidic sides of the gels are to the left and relative molecular mass declines from the top.

Separation of thyroid tissue samples from BTG (n = 20), PTCa (n = 8) and PTCb (n = 6) patients involved in the present study by 2DE generated similar profiles. An average of 758 protein spots was matched when the 2DE profiles of the patients were analyzed using ImageMaster™ 2D Platinum software. Figures 1A–1C demonstrate representative 2DE gel images of patients with BTG, PTCa and PTCb, respectively. Six protein spots, with altered abundance by more than 1.5 fold, were detected when 2DE gels of PTCa and PTCb were compared with those of BTG. Analysis by LC MS/MS Q-TOF and database query identified the proteins as alpha-1 antitrypsin (A1AT; three different protein species), heat shock 70 kDa protein (HSP70), protein disulfide isomerase (PDI) and ubiquitin-conjugating enzyme E2 N (UBE2N) (Table 1).

**Table 1:** Identification of spots from 2DE tissue protein profiles using LC MS/MS Q-TOF.

Spot no.	Tissue proteins	Swiss Prot acc. no.	Theoretical mass (Da)	MS/MS search score	Coverage (%) (number of peptide)
1	A1ATb	P01009	46,906.8	125.1	28.7 (9)
2	A1ATc	P01009	46,906.8	127.3	19.8 (9)
3	A1ATd	P01009	46,906.8	139.8	17.9 (9)
4	HSP70	P08107	70,336.2	90.8	10.2 (5)
5	PDI	P30101	57,180.7	259.9	33.8 (17)
6	UBE2N	P61088	17,194.6	44.9	19.0 (3)

**Figure 2:**
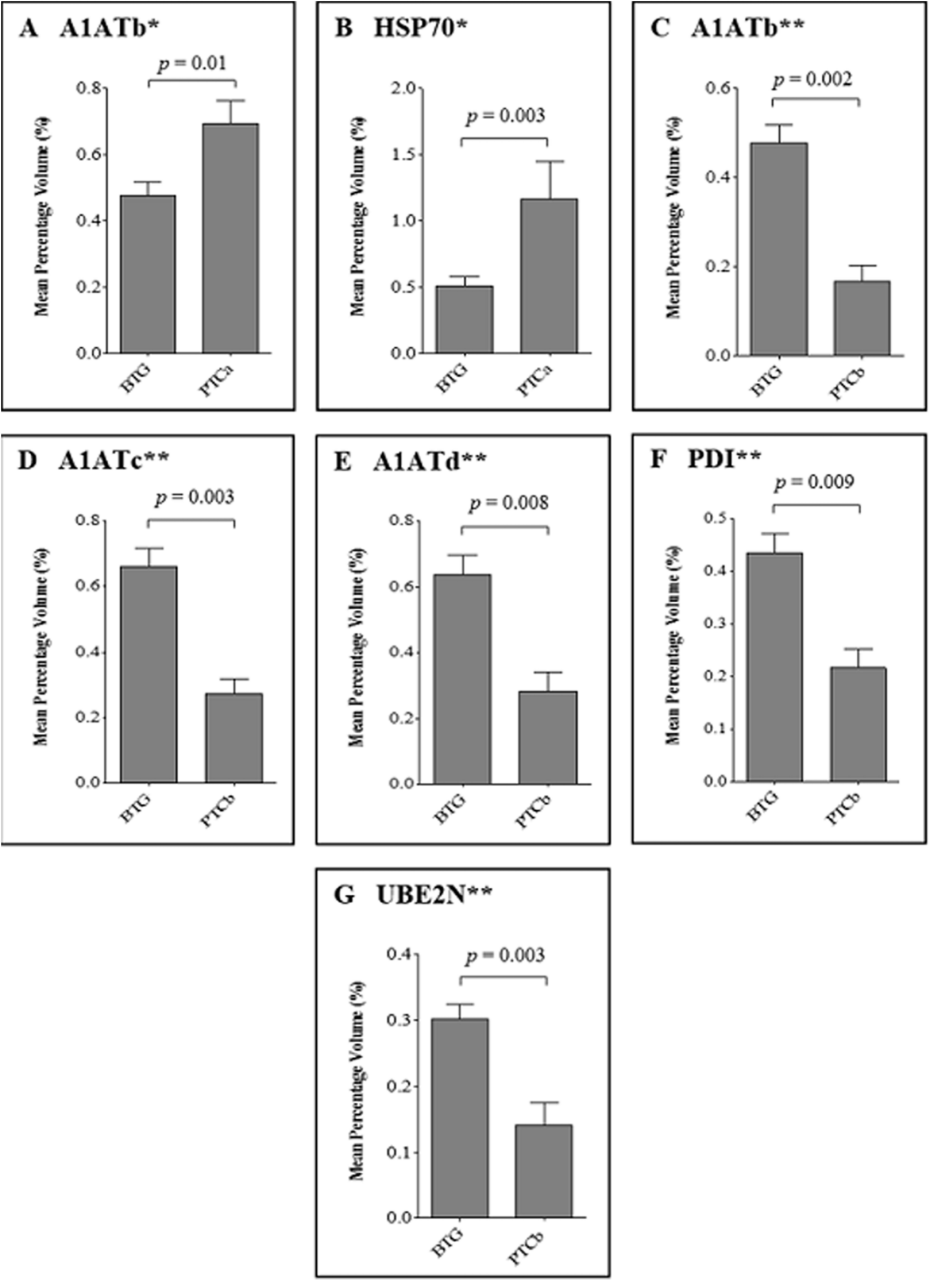
Percentage volume contribution of 2DE tissue proteins that were differentially expressed in patients with BTG, PTCa and PTCb. Percentage of volume contribution (%vol) of tissue protein spots was analyzed using ImageMaster™ 2D Platinum software, version 7.0. Standard errors of the mean (SEM) were from biological replicates. Single asterisk (*) denotes differentially expressed proteins in PTCa patients (n = 8) compared to those with BTG (n = 20) and double asterisks (**) refer to proteins that were differentially expressed in PTCb (n = 6) compared to BTG (n = 20) patients.

Figure 2 demonstrates the relative abundance of proteins that were significantly different (*p* ≤ 0.01) in the thyroid tissues of patients with PTCa (n = 8) and PTCb (n = 6) compared to those with BTG (n = 20). Two protein spots of altered abundance, including higher levels of A1ATb (*p* = 0.01; f.c. = +1.5) and HSP70 (*p* = 0.003; f.c. = +2.3) were identified in the PTCa patients compared to those with BTG (panels A and B respectively). In the case of PTCb patients, the expression of A1ATb (*p* = 0.002; f.c. = −2.8), A1ATc (*p* = 0.003; f.c. = −2.4), A1ATd (*p* = 0.008; f.c. = −2.3), PDI (*p* = 0.009; f.c. = −2.0) and UBE2N (*p* = 0.003; f.c. = −2.1) was significantly lower than those of the BTG patients (panels C, D, E, F and G, respectively).

**Figure 3:**
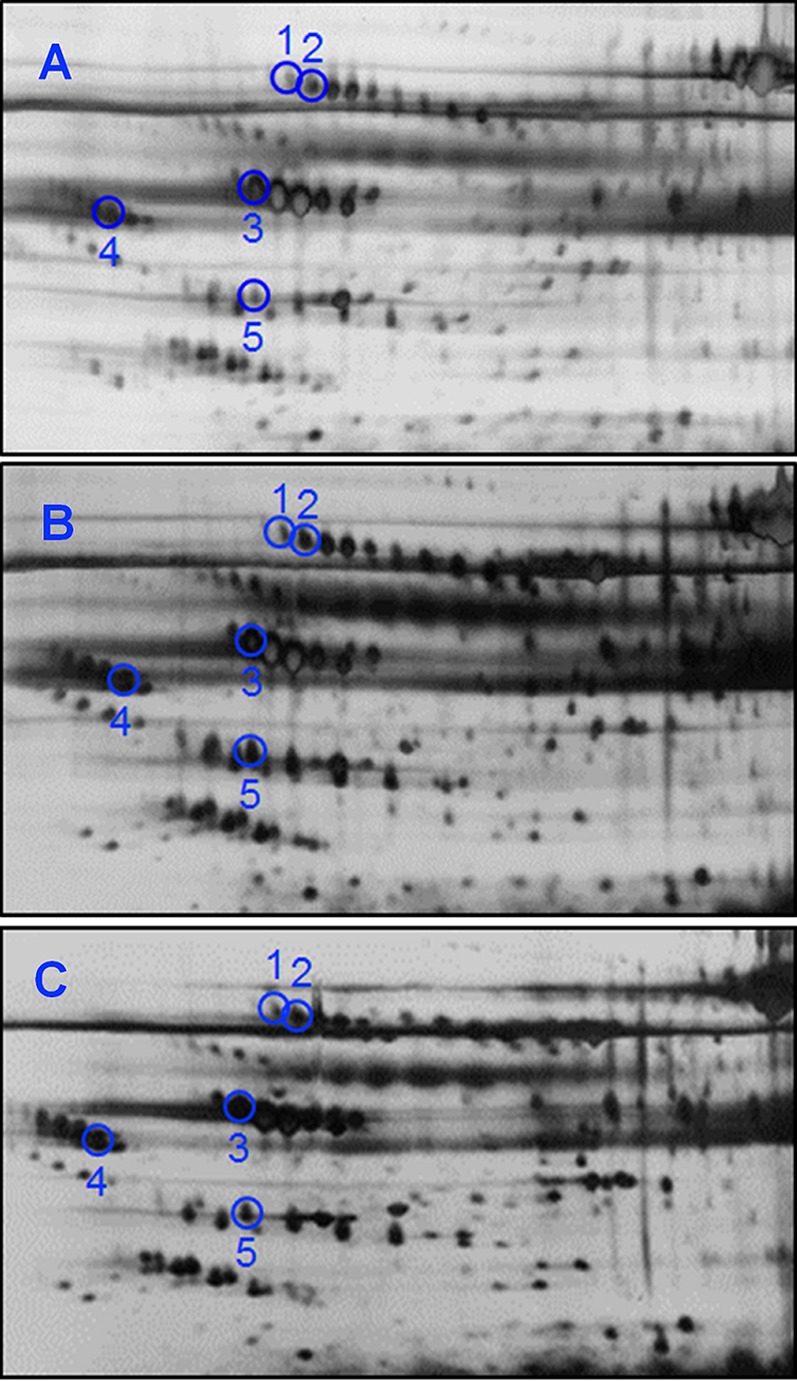
2DE analyses of serum proteins derived from BTG, PTCa and PTCb patients. Serum samples of (A) BTG (n = 20), (B) PTCa (n = 8) and (C) PTCb (n = 6) patients were subjected to 2DE and silver staining. Circles in blue refer to spots that showed differential abundance in PTCa and/or PTCb patients compared to those with BTG. The acidic sides of the 2DE gels are to the left and relative molecular mass declines from the top for all panels.

Substantially less spots were resolved when serum samples of the same patients were analyzed by 2DE. The representative 2DE serum protein profiles of patients with BTG (Panel A), PTCa (Panel B) and PTCb (Panel C) are shown in Fig. 3. An average of 377 spots was matched when a total of 34 2DE gels (BTG; n = 20; PTCa; n = 8; PTCb; n = 6) were analyzed using ImageMaster™ 2D Platinum software. Among these, five demonstrated statistically significant variation (*p* ≤ 0.01), with more than 1.5-fold difference of abundance. Five spots of significant altered abundance were detected in the 2DE profiles of PTCa or PTCb patients compared to those of patients with BTG.

**Figure 4:**
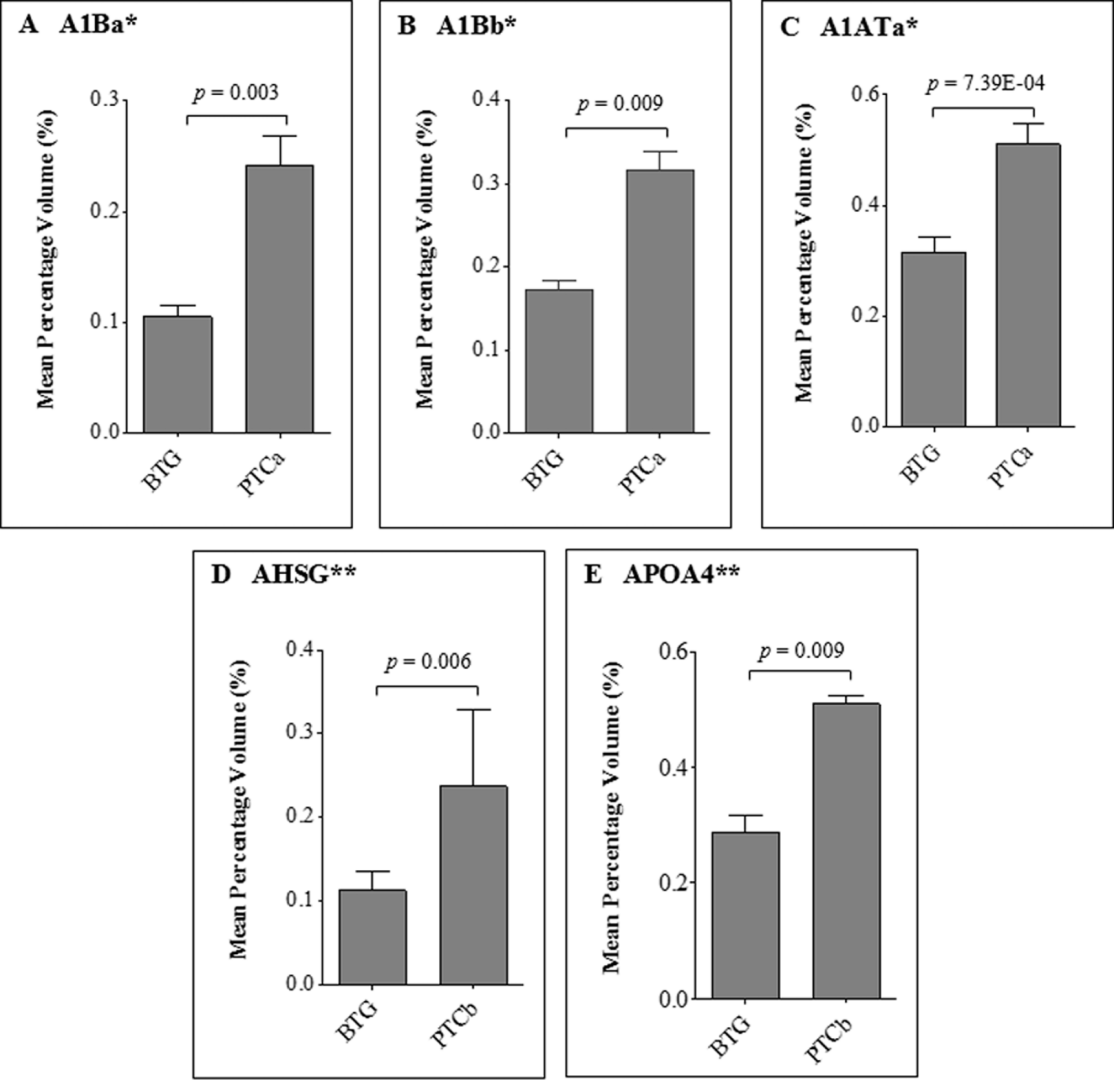
Average percentage of volumes that were differentially expressed between BTG, PTCa and PTCb patients. Percentage of volume contribution (%vol) of serum protein spots was analyzed using ImageMaster™ 2D Platinum software, version 7.0. Standard errors of the mean (SEM) were from biological replicates. Single asterisk (*) denotes differentially expressed proteins in PTCa patients (n = 8) compared to those with BTG (n = 20) and double asterisks (**) refer to proteins that were differentially expressed in PTCb (n = 6) compared to BTG (n = 20) patients.

When the five protein spots of altered abundance were subjected to LC MS/MS Q-TOF analysis and database query, three were identified as A1AT, AHSG and apolipoprotein A-IV (APOA4), whilst the other two spots were those of alpha 1-beta glycoprotein (A1B) (Table 2). Both protein species of A1B (A1Ba, *p* = 0.008; f.c.= +1.62, A1Bb, *p* = 0.003; f.c. = +1.82) and A1ATa (*p* = 7.3E-04; f.c. = +2.29) were apparently overexpressed in patients with PTCa compared to those with BTG (Fig. 4, panels A–C) whilst patients with PTCb demonstrated higher expression of AHSG (*p* = 0.006; f.c. = +2.11) and APOA4 (*p* = 0.009; f.c.= +1.77) (Fig. 4, panels D and E).

**Table 2:** List of serum proteins with differential abundance identified by LC MS/MS Q-TOF.

Spot no.	Serum proteins	Swiss Prot acc. no.	Theoretical mass (Da)	MS/MS search score	Coverage (%) (number of peptide)
1	A1Ba	P04217	54,823.0	49.7	6.8 (3)
2	A1Bb	P04217	54,823.0	140.7	24.4 (8)
3	A1ATa	P01009	46,906.8	308.5	45.2 (21)
4	AHSG	P02765	40,122.7	100.2	21.5 (6)
5	APOA4	P06727	45,371.0	61.4	10.1 (4)

**Figure 5:**
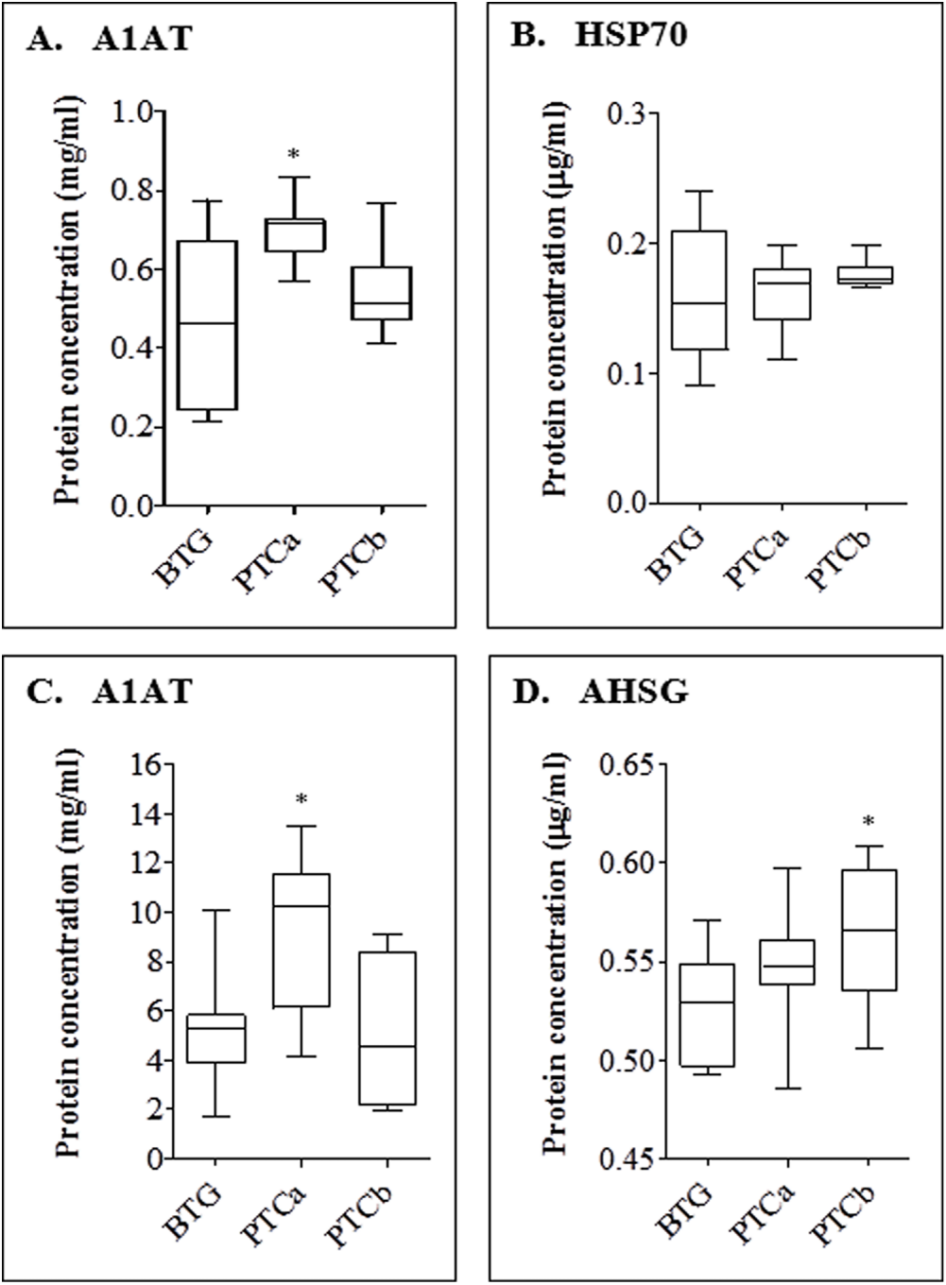
ELISA analyses of tissue (A and B) and serum (C and D) proteins in BTG, PTCa and PTCb patients. ELISA was performed using antihuman A1AT, HSP 70 and AHSG as primary antibodies. Single (*) asterisk denotes a *p* < 0.05 when PTCa (n = 8) and PTCb (n = 6) were compared to BTG (n = 20). Assay was performed in duplicate and data are presented as mean ± standard error of the mean (SEM) from biological replicates.

For confirmation of the detected proteins of altered abundances which showed more than two-fold difference (*p* < 0.01) in patients with PTCa and PTCb relative to BTG patients, ELISA was performed on the tissue and serum proteins (Fig. 5). Our results indicate that the expression of tissue A1AT was significantly higher for PTCa but not significantly different for PTCb (panel A). In case of HSP70, ELISA was not able to detect significant differences of the tissue protein in both groups of patients with PTCa and PTCb compared to those with BTG (panel B). A1AT was also found to be significantly enhanced in the sera of patients with PTCa compared to those with BTG while its levels in PTCb patients appeared lower than those with BTG (panel C). Serum AHSG was enhanced in both PTCa and PTCb patients although significant difference was only detected in patients with PTCb (panel D).

## Discussion

The association between PTCb and BTG is well recognized (Hanumanthappa et al., 2012). In Gandolfi et al. (2004) reported detection of unsuspected PTC in 14% of BTG patients after postoperative histopathologic examination, and stressed that the risk of malignancy in BTG should not be undervalued. More recently, Thavarajah & Weber (2012) performed global gene expression analysis to evaluate for dissimilarities in gene expression patterns between patients confirmed with MNG with those who had unsuspected PTC. In their report, they claimed to be able to accurately distinguish between hyperplastic nodules of the patients from those associated with PTC based on the gene expression patterns, and hypothesized factors that may influence PTC genesis. Unlike PTCb, PTCa is a PTC that has no known association with BTG. In this study, we have analyzed the thyroid tissue and serum samples of patients with BTG, PTCa and PTCb using gel-based proteomics and identified unique protein expression profiles in tissues as well as serum samples of the PTCa and PTCb patients, as compared to those with BTG. This is to identify tissue and serum proteins of altered abundance in patients with PTCa and PTCb relative to those expressed in patients with BTG.

In the first part of the study, image analysis of thyroid tissue samples resolved by 2DE demonstrated enhanced expression of A1AT and HSP70 in patients with PTCa compared to those with BTG. A1AT is an inhibitor of plasma serine proteases involved in the regulation of the activity of many serum enzymes. The protein, which shares DNA sequence homology with other members of the family of serine protease inhibitors like alpha-1 antichymotrypsin and antitrombin III, has been previously reported to be elevated in PTC compared to the benign tissues (Poblete et al., 1996). Elevated levels of HSP70 by more than two-fold difference in patients with PTC have also been previously reported by Brown et al. (2006), and this is consistent with the 2DE data of our study. However, our ELISA was not able to validate these results.

In the case of tissue samples of patients with PTCb, image analysis of 2DE gels demonstrated lower levels of A1AT, PDI and UBE2N, relative to their BTG counterparts. The lower expression of A1AT in PTCb patients appears to be in direct contrast to that detected in patients with PTCa, and hence, points to a different mechanism that may be involved in malignant transformation of PTCb as opposed to PTCa. In the present study, the different altered expression of A1AT in the tissue samples of patients with PTCa and PTCb were also validated using ELISA. To the best of our knowledge, PTCb is the only cancer whose tissue expression of A1AT is shown to be relatively lower than those in BTG tissues. An earlier study conducted by Netea-Maier et al. (2008) by examining differences of protein abundances between follicular adenoma and follicular cancer tissues has also shown a statistically significant difference in the levels of PDI, which is one of 20 proteins belonging to a family of enzymes that mediate oxidative protein folding in the endoplasmic reticulum (Kozlov et al., 2010). Similarly, UBE2N has been previously reported to be overexpressed in patients with triple-negative breast cancer as well as in six different neuroblastoma cell lines (Muñiz Lino et al., 2014; Cheng et al., 2014). The role of UBE2N in the development of neuroblastoma was explained by p53 inactivation through formation of monomeric p53 that results in its cytoplasmic translocation and subsequent loss of function (Cheng et al., 2014). This same mechanism may also occur in the development of PTCb as supported by our observation of decreased UBE2N abundance in the PTCb tissues although further investigations are needed for absolute confirmation.

When similar gel-based experiments were performed on serum samples of the same groups of PTCa, PTCb and BTG patients in the second part of the study, only A1AT was consistently detected to be of altered abundance. HSP70, PDI and UB2EN, which were earlier shown to be differently expressed in the tissue samples of patients with PTCa or PTCb, were either not detected or not significantly different when compared to patients with BTG. Among the proteins identified, A1AT demonstrated significantly higher abundance in serum samples of PTCa patients compared to those of BTG. Earlier studies have also demonstrated significantly increased levels of blood A1AT in a good number of cancers including hepatocellular carcinoma (Hong & Hong, 1991), pancreatic adenocarcinoma (Trachte et al., 2002), gastrointestinal cancers (Solakidi et al., 2004), infiltrating ductal breast carcinoma (Hamrita et al., 2010) and lung cancer (Patz et al., 2007) but the present study, performed by 2DE as well as ELISA, is the first to report on the enhanced levels of A1AT in the serum samples of PTCa patients.

A1AT is known to be present in abundance in the blood. Enhanced levels of A1AT in the blood circulation of patients with cancer are believed to be due to additional production of the protease inhibitor by the tumor cells (Poblete et al., 1996). Whilst this may be true in PTCa, it is not quite the same in the case of PTCb as our earlier analysis has shown decreased levels of A1AT in the cancer tissues of the PTCb patients. Hence, in case of the latter, enhanced abundance of serum A1AT detected is more likely to be a result of excess cell death or damage as earlier suggested by Anderson & Anderson (2002).

Aside from A1AT, elevated levels of A1B were further detected in the serum analyses of patients with PTCa. A1B, a member of the immunoglobulin superfamily, is believed to be a secreted plasma protein (Ishioka, Takahashi & Putnam, 1986). Although the function of A1B is unknown, overabundance of the protein in serum samples has been previously reported in patients with endometrial and cervical cancers (Abdul-Rahman, Lim & Hashim, 2007). In contrast to patients with PTCa, our serum analyses of PTCb patients demonstrated relatively higher levels of AHSG and APOA4 relative to those from patients with BTG. Significant higher levels of AHSG in the PTCb patients relative to those with BTG were also detected in our ELISA experiments. Interestingly, the levels of APOA4 have been similarly shown to be able to discriminate malignant from benign cases in ovarian and endometrial neoplasms (Wang et al., 2011; Timms et al., 2014). However, in the case of AHSG, which is a major growth promoter in serum (Kundranda et al., 2005), earlier studies have reported its reduced abundance in patients with acute myeloid leukemia (Kwak et al., 2004), lung squamous cell carcinoma (Dowling et al., 2007) and germ-line ovarian carcinoma (Chen et al., 2008).

When taken together, the data of our tissue and serum analyses of PTCa and PTCb patients relative to those with BTG generally points to differences in the two distinct cancers of the thyroid although it is currently not possible to rule out that they may also be due other reasons such as the different stages of the malignant disease. Both PTCa and PTCb distinguish themselves from BTG in different types of tissue and serum proteins of altered abundance. While lower levels of tissue A1AT, PDI and UBE2N appeared to be associated with PTCb, higher abundances of A1AT and HSP70 seemed apparent in PTCa. In case of the serum proteins, higher abundances of A1AT and A1B were detected in PTCa, while PTCb was associated with enhanced APOA4 and AHSG. Because of their distinctive altered abundances, these proteins also stand to have potential use as tissue or serum biomarkers for distinguishing the three different thyroid neoplasms although this requires further extensive validation in clinically representative populations. In our recent case study report, we have shown that the benign and malignant tissues of a patient with concurrent benign thyroid cyst and PTC were both detected for the c.353G > A (p.R118Q) mutation of the TRH gene but only the malignant tissue showed presence of the c.1799T > A (p.V600E) mutation in the BRAF gene (Lee et al., in press). This patient had five years earlier presented with BTG. Data generated from proteomics analysis of the tissues led us to the speculation that the malignancy in the patient may have been due to BRAF^V600E^ mutation that was initiated from prolonged H_2_O_2_ insults attributed to the germ line TRH^R118Q^ mutation. Whilst this may be the molecular basis for malignancy in patients with PTCb, different mechanisms, such as germ line or direct mutation of the BRAF gene (Waltz et al., 2014; Yu et al., 2015), may have been implicated in those with PTCa.

## Supplemental Information

10.7717/peerj.2450/supp-1 Supplemental Information 1 Percentage volume contribution of 2DE tissue proteins that were differentially expressed in patients with BTG, PTCa and PTCb. (A) Shows the anova value given by the ImageMaster 2D Platium v 7.0 software and details of the percentage of volume contribution of A1ATb; (B) Shows the anova value given by the ImageMaster 2D Platium v 7.0 software and details of the percentage of volume contribution of HSP70; (C) Shows the anova value given by the ImageMaster 2D Platium v 7.0 software and details of the percentage of volume contribution of A1ATb; (D) Shows the anova value given by the ImageMaster 2D Platium v 7.0 software and details of the percentage of volume contribution of A1ATc; (E) Shows the anova value given by the ImageMaster 2D Platium v 7.0 software and details of the percentage of volume contribution of A1ATd; (F) Shows the anova value given by the ImageMaster 2D Platium v 7.0 software and details of the percentage of volume contribution of PDI; (G) Shows the anova value given by the ImageMaster 2D Platium v 7.0 software and details of the percentage of volume contribution of UBE2N.

10.7717/peerj.2450/supp-2 Supplemental Information 2 Percentage volume contribution of 2DE serum proteins that were differentially expressed in patients with BTG, PTCa and PTCb. (A) Shows the anova value given by the ImageMaster 2D Platium v 7.0 software and details of the percentage of volume contribution of A1Ba; (B) Shows the anova value given by the ImageMaster 2D Platium v 7.0 software and details of the percentage of volume contribution of A1Bb; (C) Shows the anova value given by the ImageMaster 2D Platium v 7.0 software and details of the percentage of volume contribution of A1ATa; (D) Shows the anova value given by the ImageMaster 2D Platium v 7.0 software and details of the percentage of volume contribution of AHSG; (E) Shows the anova value given by the ImageMaster 2D Platium v 7.0 software and details of the percentage of volume contribution of APOA4.

10.7717/peerj.2450/supp-3 Supplemental Information 3 ELISA analyses of tissue (A and B) and serum (C and D) proteins in BTG, PTCa and PTCb patients. (A) Shows absorbance value and protein concentration of tissue A1AT; (B) Shows absorbance value and protein concentration of tissue HSP70; (C) Shows absorbance value and protein concentration of serum A1AT; (D) Shows absorbance value and protein concentration of serum AHSG.

## Additional Information and Declarations

### Data Deposition

The following information was supplied regarding data availability: The raw data has been supplied as Supplemental Dataset Files.
